# Cassava whitefly, *Bemisia tabaci* (Gennadius) (Hemiptera: Aleyrodidae) in East African farming landscapes: a review of the factors determining abundance

**DOI:** 10.1017/S0007485318000032

**Published:** 2018-02-13

**Authors:** S. Macfadyen, C. Paull, L.M. Boykin, P. De Barro, M.N. Maruthi, M. Otim, A. Kalyebi, D.G. Vassão, P. Sseruwagi, W.T. Tay, H. Delatte, Z. Seguni, J. Colvin, C.A. Omongo

**Affiliations:** 1 CSIRO, Clunies Ross St. Acton, ACT, 2601, Australia; 2 CSIRO, Boggo Rd. Dutton Park, QLD, 4001, Australia; 3 University of Western Australia, School of Molecular Sciences, 35 Stirling Highway, Crawley, WA 6009, Australia; 4 Natural Resources Institute, University of Greenwich, Chatham Maritime, Kent, ME4 4TB, UK; 5 National Crops Resources Research Institute, Kampala, Uganda; 6 Mikocheni Agricultural Research Institute, P.O. Box 6226 Dar es Salaam, Tanzania; 7 Max Planck Institute for Chemical Ecology, Hans-Knoell Str. 8 D-07745 Jena, Germany; 8 CIRAD, UMR PVBMT, Saint Pierre, La Réunion 97410-F, France

**Keywords:** cassava, ecology, natural enemies, climate change, cultivars

## Abstract

*Bemisia tabaci* (Gennadius) (Hemiptera: Aleyrodidae) is a pest species complex that causes widespread damage to cassava, a staple food crop for millions of households in East Africa. Species in the complex cause direct feeding damage to cassava and are the vectors of multiple plant viruses. Whilst significant work has gone into developing virus-resistant cassava cultivars, there has been little research effort aimed at understanding the ecology of these insect vectors. Here we assess critically the knowledge base relating to factors that may lead to high population densities of sub-Saharan African (SSA) *B. tabaci* species in cassava production landscapes of East Africa. We focus first on empirical studies that have examined biotic or abiotic factors that may lead to high populations. We then identify knowledge gaps that need to be filled to deliver sustainable management solutions. We found that whilst many hypotheses have been put forward to explain the increases in abundance witnessed since the early 1990s, there are little published data and these tend to have been collected in a piecemeal manner. The most critical knowledge gaps identified were: (i) understanding how cassava cultivars and alternative host plants impact population dynamics and natural enemies; (ii) the impact of natural enemies in terms of reducing the frequency of outbreaks and (iii) the use and management of insecticides to delay the development of resistance. In addition, there are several fundamental methodologies that need to be developed and deployed in East Africa to address some of the more challenging knowledge gaps.

## Introduction

*Bemisia tabaci* (Gennadius) (Hemiptera: Aleyrodidae) is a pest species complex that causes widespread damage to cassava, a staple food crop in many millions of smallholder households in Africa (Otim-Nape *et al*., 2000; Colvin *et al*., 2004; Legg *et al*., 2006; Patil *et al*., 2015). *Bemisia tabaci* causes direct feeding damage to cassava, excretes a sugar-rich honeydew, which acts as a substrate for sooty moulds that reduces both respiration and photosynthesis (Nelson, 2008). In addition, *B. tabaci* vector multiples plant viruses that cause two damaging diseases: cassava mosaic disease (CMD) and cassava brown streak disease (CBSD), that in combination lead to significant yield loss in cassava (Holt & Colvin, 2001; Maruthi *et al*., 2002a, b). Whilst substantial effort has gone into developing virus-resistant cassava cultivars, there has been little research effort aimed at understanding this insect vector, which alone can reduce yields, by 40% (Thresh *et al*., 1997). This disproportionate approach to managing insect-vectored plant diseases is not unusual, but has led repeatedly to management solutions that are not sustainable. Based on partial mtCO1 gene sequence phylogenetic analysis, the *B. tabaci* complex is composed of four major clades (a clade is a group of organisms believed to have all descended from a common ancestor). The sub-Saharan Africa (SSA) clade forms the ancestral root (Boykin *et al*., 2013) of the complex, and in recent history, species in this clade have been associated with an increased frequency of cassava viral disease outbreaks in East Africa. This review of the empirical evidence is timely and necessary as we need to identify clearly the biotic and abiotic factors that may have contributed to high population growth of *B. tabaci* in the past, before we can develop urgently needed and sustainable management recommendations for the future.

Whilst many hypotheses have been put forward about the factors that may be contributing to high *B. tabaci* populations on cassava in East Africa, there are little data available and these tend to have been collected in a piecemeal manner.

Our objectives for this review are firstly, to synthesize the existing literature on the SSA *B. tabaci* species’ ecology in East Africa and to review critically this knowledge base. We focused on empirical studies that have examined factors that may lead to high populations or outbreaks of the SSA *B. tabaci*. We then identified the gaps in knowledge and understanding that need to be filled to deliver long-term sustainable solutions to manage both the vector species and the viruses that they transmit. Westarted by listing factors that, from an *a priori* perspective, are likely to be important ecological determinants of *B. tabaci* abundance (table 1) in any farming context. Factors that may support or limit population growth were equally considered (as these both may facilitate outbreaks). We then searched for studies based in East African production landscapes, preferring those focused on cassava. We included the countries of Tanzania, Uganda, Rwanda, Burundi, South Sudan and Malawi as part of the geographical region of Eastern Africa. In cases where we could not find published studies based in East Africa, we cited geographically related work if relevant. We excluded studies that look solely at virus impacts on the crop, and there have been several important review articles that have summarized information on cassava virus disease epidemics and speculated on some of the likely causes (table 2). In addition, there are reviews by Fishpool & Burban (1994); Legg (1994) and Colvin *et al*. (2006) that provide a good baseline of ecological and biological information on what was known about *B. tabaci* complex and cassava viruses up until the late 1990s. A complicating factor in reviewing the evidence base for factors relating to East African *B. tabaci* is that our understanding of *B. tabaci* as a species has changed in the previous decade and so it is at times unclear as to the actual identity (as determined by their partial mtCO1 gene sequence) of the species being referred to, especially in older references. Where possible, we attempted to resolve these issues.

**Table 1:** Potential factors influencing *Bemisia tabaci* abundance on cassava included in this review (does not include interactions between these factors). We have suggested the likely direction of the effect in terms of an increase (↑) or decrease (↓) in *B. tabaci* abundance, but note there are many possible outcomes for some of these factors.

Factors	Potential mechanisms that may lead to a change in abundance	Likely direction of effect
Cassava cultivar	Leaf architecture (e.g. width of leaves) Growth habit (e.g. long vs. short growing season) Plant chemistry differences between cultivars	Wider leaves = ↑ nymph density ↑ Up to *6 months after planting, then steady ↓. Exact cause unknown Unknown – depends on compounds involved
Cassava age	Number of new leaves at the top of the plant Change in plant chemistry as cassava ages	More new leaves = ↑ adult density Unknown – depends on compounds involved
Infection status of cassava	Fecundity and survivorship enhanced on infected hosts Promotion of emigration of *B. tabaci* adults	↑ Adult and nymph density on cassava plants Unknown – may increase populations, but also spread densities
Non-cassava host plants	Other crops, natural vegetation andweeds act as host plants for *B. tabaci*	↑ Population density in cassava if more resources present in landscape year-round
Spatial arrangement and amount of host plants surrounding cassava fields	More resources for *B. tabaci* at important timesMore resources for natural enemies	↑ Population density in cassava if more resources present in landscape year-round
		↓ Population density in cassava if more resources for naturalenemies present in landscape year-round
Natural enemies	Predators consuming *B. tabaci* Parasitoids using *B. tabaci* as host	↓ Nymph density from increased mortality due to natural enemies Unknown – intra-guild predation effects
Other pests on cassava	Cassava green mite damage to top leaves. Reduces suitable space on plant for *B. tabaci* adults	↓ Adult density on top leaves may lead to reduced oviposition Unknown – synergistic effects of multiple pests overcoming host-plant defences
Endosymbionts	Presence of some endosymbiont species in *B. tabaci* can decrease the number of adults emerging, increase development time, thus reducing overall population development	↓ Healthy adult emergence rate ↓ Population density in landscape
Altitude	Unclear, combination of temperature, rainfall and host-plant availability. Less suitable conditions at higher altitudes	Unknown
Climate	Long-term changes in temperature and rainfall	Unknown
Weather	Heavy rainfall events Very high temperatures	↓ Nymph density, through increasing mortality due to heat stress and dislodgement ↓ Population density perhaps through disrupting adult behaviour
Pesticides	Resistance in *B. tabaci* Pesticides killing natural enemies or competitors	↑ Population density in landscape
New invasive species in East Africa	Totally newspecies has taken over from local species in cassava (species turnover)	Unknown. It is unclear how this would lead to a change in abundance in isolation from other factors
Hybridization	‘Invader biotype’ out-competes domestic species and is better able to use resources	Unknown. It is unclear how this would lead to a change in abundance in isolation from other factors

**Table 2:** Review articles with relevant information about *Bemisia tabaci* biology and ecology.

Citation	Topics covered
Legg *et al*. (2014b)	Historical account of virus outbreaks Emergence of ‘superabundant’ *B. tabaci* Control options for *B. tabaci*
Legg *et al*. (2011)	Regional epidemiology of cassava virus pandemics across Africa Comparison of characteristics of CMD and CBSD outbreaks
Patil & Fauquet (2010)	CMBs, knowledge and perspectives Very comprehensive review of the cassava viruses
Legg & Thresh (2000)	CMD dynamics in East Africa Mechanisms behind the spread of the CMD pandemic
Legg (1999)	Describes the pandemic of CMD across east and central Africa Strategies to control the pandemic
Otim-Nape *et al*. (1995)	*B. tabaci* and CMD in Africa Very comprehensive treatment of all aspects of the disease and vector story
Fishpool & Burban (1994)	Biology of *B. tabaci* including morphology, taxonomy, bionomics Ecology on cassava in Africa Some discussion about natural enemies and control
Legg (1994)	Ecology of whitefly and CMBs pathosystem Factors affecting population development of *B. tabaci*; temperature, climate, rainfall, host-plant chemistry, architecture and age, natural enemies Interactions between *B. tabaci* and other cassava pests

CMBs, cassava mosaic begomoviruses; CMD, cassava mosaic disease; CBSD, cassava brown streak disease.

### African *B. tabaci* species complex: naming and identification

Throughout this review, we use *B. tabaci* to mean the *B. tabaci* species complex found in East Africa. However, the identification of the species involved in these outbreaks based on genetic differences has only recently been attempted (see example from Kenya in Manani *et al*., 2017). Due to morphological similarities, *B. tabaci* was originally thought to be one species worldwide, but based on genetic differences (Colvin *et al*., 2004; Sseruwagi *et al*., 2005; Boykin *et al*., 2007; 2013; Wang *et al*., 2014); and mating incompatibility (Colvin *et al*., 2004; Xu *et al*., 2010; Liu *et al*., 2012), it is now recognized as a species complex with at least 34–36 species (Boykin *et al*., 2012; Barbosa *et al*., 2015). This discovery of further species diversity has led to many nomenclatural changes over the last 10 years causing confusion in the literature (Boykin & De Barro, 2014; Boykin *et al.*, 2018).

The SSA *B. tabaci* species are no exception to the nomenclatural confusion. Identification of species in the *B. tabaci* pest complex currently relies on the 3’ region of 657 bp partial mt DNA COI gene identity. However, many names have been used for the same SSA entities with little consistency from study to study. The naming confusion has made it difficult to compare studies of ecological importance across time or fromdifferent researchers. For example, Sseruwagi (2005) used ‘Ug1’, Legg *et al*. (2014a) used ‘SSA1 subgroups 1–3’ and Mugerwa *et al*. (2012) used ‘SSA1 subclades I–III’ based on mtCO1 data. Are these the same entity? In short, no. Relevant to this study are the SSA1 and SSA2 species of *B. tabaci*, where Ug1 = SSA1 and further subdivisions of that species include SSA1 subgroup 1 (Legg *et al*., 2014a) = SSA1 subclade I (Mugerwa *et al*., 2012). However, Ug2 (Sseruwagi *et al*., 2005) translates directly to SSA2 (Mugerwa *et al*., 2012; Legg *et al*., 2014a) with little confusion. Most of the confusion involves the SSA1 species, because most studies did not compare their SSA1 mtCO1 sequences against the then known available diversity. This meant that their data were not set firmly within a complete understanding of *B. tabaci* diversity at the time (Boykin *et al.*, 2018).

Greater clarity around the species identity of individuals involved in future outbreaks may help to uncover the causes of these outbreaks. Even closely related species may differ in their host-plant use, ability to transmit viruses, fecundity and response to management actions. Conclusions and findings from past work in this region, however, are still useful to understanding the ecology of the species complex. In addition, species-specific management strategies and interventions could play a larger role in the future (see ‘Knowledge gaps’ section towards the end of this review).

### Overview of the life cycle of *B. tabaci*

The life-history parameters of many species in the *B. tabaci* complex vary depending on the environmental conditions and the host plant they develop on. The published information suggests that the development period of *B. tabaci* from egg to adult emergence is between 19 and 29 days, and the species goes through four nymphal instars before entering a pupal phase (Colvin *et al*., 2006). Depending on the environmental conditions, there can be 11–12 generations of *B. tabaci* per year (Asiimwe *et al*., 2007a; b). In East Africa, cassava is planted from cuttings twice per year in some parts of Uganda, through to one cropping season in Malawi. Depending on the cultivar used, the plant can remain in the ground for 6–12 months before the tuber is ready to be harvested. Often, cassava is planted in a mixed field with maize, coffee and banana, and multiple cassava fields of different ages can exist in one location, providing year-round host plants for *B. tabaci*. A description of the different developmental stages of *B. tabaci* on cassava, using a colony established in Uganda, is presented in Thompson (2000). Adult female *B. tabaci* produce 4–5 eggs per day and these are oviposited on the underside of the leaves and the leaf petiole. Both the adults and nymphs have sucking mouthparts to pierce the leaf tissue and consume phloem sap. Adults prefer to congregate and alight on the immature upper leaves of the cassava plant (Sseruwagi *et al*., 2004). The first nymphal stage is mobile until it finds a suitable feeding location. The nymphs exude honeydew, which falls onto the lower leaves of the plant leading to sooty mould development.

There are a range of abiotic and biotic factors (e. g. host-plant availability, weather, mortality from natural enemies, etc. ) that may influence the abundance of any pest herbivore on a host plant. Understanding how these factors relate to population dynamics and distributions measured at the field level and scale-up to the regional level is critical for determining if a pest outbreak is likely to occur. We define an outbreak situation as one in which the pest herbivore or plant-virus vector has been released from control, has reached high abundances, and is causing economic injury to the crop. This problem usually manifests at the field or regional scale. Importantly, crop damage can occur at low pest abundance, especially in the case of virus transmission. Thus, whilst outbreaks are often obvious to farmers and the general community, significant yield loss and damage can occur in non-outbreak situations. Here we focus on the documented evidence of factors that influence abundance of *B. tabaci* on cassava in East Africa. There are likely to be a number of factors that will, in isolation or in combination, influence the abundance of *B. tabaci* in cassava landscapes. We have classified these into biotic (cassava cultivar, cassava age, cassava virus infection status, non-cassava host plants, natural enemies, competition with other herbivores and endosymbionts), abiotic (altitude, climate and weather) and other factors (pesticides, hybridization) in table 1.

### History of *B. tabaci* abundance on cassava and outbreaks in East Africa

**Fig. 1:**
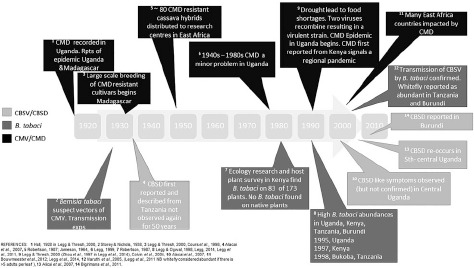
Timeline of events of *Bemisia tabaci* and associated disease outbreaks in East Africa. CMV, cassava mosaic virus; CMD, cassava mosaic disease; CBSV, cassava brown streak virus, CBSD, cassava brown steak disease.

There has been a change in the abundance of *B. tabaci* in cassava production landscapes in East Africa in general over time (fig. 1). However, quantitative definitions of what is a high or low population abundance have also changed across time; therefore, empirical evidence documenting this change is weak. The threshold of the number of adults considered highly abundant, however, differs between studies, and we cannot translate abundance data into likely yield loss. Early research from Ivory Coast considered cassava a poor host for *B. tabaci*, as numbers rarely exceeded 300 adults per plant and more often there were 150 adults per plant (Fishpool & Burban, 1994; Fishpool *et al.*, 1995; Colvin *et al.*, 1998;). However, other researchers might consider these to be relatively high numbers. In Legg et al. (2011) when >5 adults per top five leaves per plant were recorded, this was considered highly abundant. In contrast, Omongo et al. (2012) only considered populations >20 adults per top five leaves per plant as high. Some quantitative studies have been summarized in table 3; however, it is still challenging to compare across studies that have used different sampling methodologies to document overall trends. Sseruwagi et al. (2004) provides a summary of mean number of *B. tabaci* from top five leaves from African studies prior to 2004.

**Table 3:** Studies quantifying the mean number of adults (unless otherwise mentioned) *Bemisia tabaci* on cassava. General method used was counting the numbers of adults observed on the top five expanded leaves on 30 plants per field and on cassava aged 3–6 months after planting (Sseruwagi *et al.*, 2004). There was some variation in methods between studies.

Mean count of *B. tabaci*	Country	Citation
Max. ~ 30 (method not confirmed)	Ivory Coast	Fargette *et al.* (1985)
Max. ~25	Ivory Coast	Fargette *et al.* (1988)
Min. ~3		
Max. ~ 35 intercropped low maize density		
Min. ~ 2. 5 intercropped low maize density (method not confirmed)		
Max. ~ 18 intercropped high maize density		
Min. ~ 2 intercropped high maize density		
Max. 14 Min. 2. 4	Ivory Coast	Fauquet *et al.* (1988)
Max. ~ 35	Ivory Coast	Fargette *et al.* (1990)
4. 6 ± 0. 54 adults and 43 ± 6. 0 nymphs (cassava no mosaic disease symptoms)	Uganda	Gibson *et al.* (1996)
5. 0 ± 0. 38 adults and 46 ± 6. 4 nymphs (cassava with mosaic disease symptoms)		
Max. 21. 8	Zambia	Muimba-Kankolongo *et al.* (1997)
Min. 0. 2		
Max. 3. 7	Uganda	Legg & Ogwal (1998)
Min. 0. 3		
>3 per shoot (three districts)	Uganda	Otim-Nape *et al.* (2001)
>1 (14 districts)		
between 1-3 (ten districts)		
(One shoot = top four expanded leaves)		
Max ~ 37	Uganda	Colvin *et al.* (2004)
Min. ~ 1		
3-4 instar nymphs = 35. 8 (early season) 59. 1 (late season) resistant cultivars	Uganda	Otim *et al.* (2006)
3-4 instar nymphs = 17. 2 (early season) 31. 2 (late season) susceptible cultivars		
Nymphs 11. 81 ± 0. 84 improved cultivars	Rwanda	Night *et al.* (2011)
Nymphs 4. 30 ± 0. 12 local cultivars		
2. 12 ± 0. 17 improved cultivars		
0. 60 ± 0. 03 local cultivar		
0. 74 ± 0. 03 inter-cropped cassava		
0. 94 ± 0. 07 mono-cropped cassava		
Max. 39.2 ± 4.4 cultivar TMS I92/0067	Uganda	Omongo *et al.* (2012)
Min. 5.4 ±1.7 cultivar Njule Red		
Max. 2. 12	Zambia	Chikoti *et al.* (2013)
Min. 0. 02		
Max. 76	Tanzania	Tajebe *et al.* (2015)
Min. <1		
Max. 1. 35	Tanzania	Jeremiah *et al.* (2015)
Min. 0. 1		
Max. 50	Malawi	Mbewe *et al.* (2015)
Min. 0		
Max. 41. 5	Tanzania	Uzokwe *et al.* (2016)
Min. 1. 0		

We have summarized the available evidence on the historical outbreaks of B. tabaci, and the two major diseases of cassava, CMD and CBSD, across East African countries in fig. 1. There are records of high populations of *B. tabaci* causing problems for farmers since the 1990s. As with most pest outbreaks, there is a focus on data collection and analysis during the outbreak phase, until an intervention (e.g. the introduction of new cassava cultivars) or change in the environment stops the outbreak, but a lack of information in the intervening periods. This makes it challenging to assess the causes and frequency of outbreaks, both at the local level and across the East African region. It is notable that the movement of infected cuttings (between regions within countries, and between countries) was implicated in a number of historical outbreaks (Alicai *et al.*, 2007). Importantly, the introduction and dissemination of new CMD-resistant cultivars to combat food shortages because of epidemics was also facilitated through these routes. Less well documented is that disease sources can be present in endemic host plants such as Jatropha sp., and trade routes between India and Africa may have also facilitated disease spread (Swanson & Harrison, 1994).

### Plant virus transmission by *B. tabaci*

Outbreaks of CMD, which are at least partially whitefly-borne, have been occurring in East Africa since the 1960s (Jameson, 1964). A detailed description of both CMD and CBSD can be found in Mabasa (2007), but we will summarize some of the key points here. There are seven cassava mosaic begomoviruses (CMBs) (Geminiviridae; genus *Begomovirus*) that are related to CMD (Legg *et al.*, 2015). The first widespread outbreaks of CMD were reported in the 1930s in East Africa (Storey & Nichols, 1938; fig. 1) and the presence of CMD is now confirmed in cassava across East Africa. CMBs appear to be persistent in B. tabaci; however, there may be some co-adaptation between the viruses and different vector species that alter their ability to transmit virus to cassava (see Maruthi *et al.*, 2002b). Severe infection causes stunting of shoots, leaves and stems which reduce tuber growth and subsequently yield (Fauquet & Fargette, 1990; Maruthi *et al.*, 2002a, b; Omongo, 2003). There is a latent period after the first leaves appear of about 1 month between time of infection by *B. tabaci* and CMD symptom expression in cassava (Fauquet & Fargette, 1990). Symptoms increase until approximately 60 days after planting. However, infection introduced beyond 5 months after planting (MAP) via *B. tabaci* has very little impact on the yield. This is because at five MAP, the tubers have started to form and the plant is still able to provide significant yield (Fargette *et al.*, 1990).

The second major cassava plant disease associated with *B. tabaci* is CBSD. CBSD is often found together with CMD, but this was not always the case (Alicai *et al.*, 2007). Historically, CBSD was thought to be caused by two distinct viruses, cassava brown streak virus (CBSV) and *Ugandan cassava brown streak virus* (UCBSV), but Ndunguru et al. (2015) have recently found more genetic diversity in both CBSV and UCBSV, suggesting that there may be more than two viruses involved. Both virus groups belong to the genus *Ipomovirus*, and family Potyviridae (Mbewe *et al.*, 2015); however, CBSV has a five times faster rate of evolution, and is more virulent compared with UCBSV (Alicai *et al.*, 2016). Unlike CMBs, CBSVs are semi-persistent in *B. tabaci* (Maruthi *et al.*, 2005). Symptoms of CBSD include yellow blotchy patches on the leaves and a change in the colour of the leaf veins, especially on the lower more mature leaves. Brown coloured vertical lesions occur on the stems and roots can become contorted and constricted. Cross-sections of roots from infected cassava plants show brown necrotic tissue (Nichols, 1950; Hillocks & Jennings, 2003; Ntawuruhunga & Legg, 2007).

*Bemisia tabaci* species can carry and potentially transmit hundreds of different plant viruses (Morales & Jones, 2004; Polston *et al.*, 2014). Harrison et al. (1997) makes the argument that selection and subsequent spread of viruses by certain *B. tabaci* species might be possible. Different species of *B. tabaci* are believed to be able to transmit Geminiviruses with different coat proteins (McGrath & Harrison, 1995; Maruthi *et al.*, 2002a; Morales & Jones, 2004, b). This may be important; how ever, methods to test for these synergistic virus-vector relationships are rare (Patil & Fauquet, 2010). Both CMD and CBSD are spread through the propagation of infected cassava cuttings and vectored by *B. tabaci* in East Africa (Maruthi *et al.*, 2005; Jeremiah *et al.*, 2014, confirmed *B. tabaci* transmits CBSVs). Transmission of CMBs by *B. tabaci* has been confirmed in Africa (Burban *et al.*, 1992; Fishpool & Burban, 1994; Gibson *et al.*, 1996; Legg *et al.*, 2002; Antony *et al.*, 2006). Survey of cassava across Tanzania during the 1993–1994 growing season showed that on average 27% of plants had CMD symptoms, of which 3% could be attributed to *B. tabaci* transmission, compared with 24% of infections to the use of infected cuttings (Legg & Raya, 1998). More recently, it has been shown that a greater proportion of CMD is cutting borne compared with being vectored by *B. tabaci* (Night *et al.*, 2011). A modelling study exploring CBSD spread showed that in a scenario with whitefly dispersal alone, large-scale epidemics were less likely than when trade of infected cuttings is also included in the model (McQuaid *et al.*, 2017). Research by Dubern (1994) indicated that *B. tabaci* was not an efficient vector of CMBs. However, Maruthi et al. (2002a, b) used CMB isolates and *B. tabaci* sourced from four different areas (three African locations and one culture from India) to show that African CMBs were transmitted by African *B. tabaci* to 60-79% of the cassava plants. However, inoculation was significantly less when Indian *B. tabaci* transmitted an African CMD isolate and *vice versa* when *B. tabaci* from Tanzania transmitted CMB isolates from India. These results were used to support the idea that there is virus and or vector co-adaptation and that there is variability in vector competence and biological traits between *B. tabaci* species (Maruthi *et al.*, 2002b). However, there is little quantifiable evidence for this hypothesis, and what evidence there is has been drawn from data that have a small number of samples (Xu *et al.*, 2010).

### Factors influencing *B. tabaci* abundance

Below we summarized the available evidence that may demonstrate a link with each factor and change in abundance of *B. tabaci* populations.

### Biotic factors

#### Cassava cultivar effects

The primary way to manage disease in cassava has been to develop cultivars that are disease resistant or tolerant (these are often referred to as ‘improved cultivars’). Observations that some cultivars were susceptible to disease have been evident since the first outbreak of CMD in the 1930s (Storey & Nichols, 1938). The key response to the 1990s CMD epidemic was to distribute cassava cuttings from improved cultivars (Oliveira *et al.*, 2001). In recent times, greater numbers of adult B. tabaci, and sometimes nymphs, have been associated with recently developed cultivars, although the dynamics of *B. tabaci* populations in semi-field situations have not been well documented (e.g. Katono *et al.*, 2015). Severity of cassava green mite (CGM; Mononychellus tanajoa) and CMD were higher on local cultivars of cassava, although *B. tabaci* populations were higher on improved cultivars (Night *et al.*, 2011). To determine which cultivars showed some level of phenotypic resistance or tolerance to B. tabaci, 19 cultivars were exposed to *B. tabaci* for colonization. Numbers of nymphs, eggs, damage and sooty mould were greatest for cultivar I92/0067 and least for Njule Red (a local cultivar) (Omongo *et al.*, 2012). Cassava leaf area did affect the severity of sooty mould (i.e. a cultivar with a lower number of *B. tabaci* could have a higher sooty mould severity score, presumably due to broader leaves). However, there was no obvious correlation between the numbers of *B. tabaci* adults and cultivar plant traits such as leaf width or colour (Omongo *et al.*, 2012).

Beyond the obvious differences in plant morphology seen between different cassava cultivars, plant biochemistry may also play a role in determining suitability for growth and development of *B. tabaci* populations. Research on the phyto-chemistry of cassava has largely concentrated on defensive metabolites such as flavonoids, hydroxycoumarins, terpenoids and cyanogenic glucosides and their distribution within plant tissue. This work was recently reviewed by Blagbrough et al. (2010). Cassava phytochemistry can impact phloem feeders, with examples including the effect of its flavonoids and cyanogenic glucosides on the cassava mealybug, *Phenacoccus manihoti* (Calatayud *et al.*, 1994a, b, 1997) and the cassava hem- ipteran pest, Cyrtomenus bergi (Riis *et al.*, 2003). *Bemisia tabaci* can also be affected, and has been shown to induce cyanide metabolizing enzymes when feeding on cassava compared with sweet potato (Antony *et al.*, 2006). These results provide evidence that defensive plant metabolites play an important role in cassava colonization by phloem feeders including *B. tabaci*. However, how the phytochemistry of different cassava cultivars and tissues influences *B. tabaci* resistance remains unknown. Future efforts should be directed at confirming these mechanisms and explaining the effect of cassava plant chemistry on phloem feeders and other herbivores within the East African cassava environment.

#### Cassava age

As cassava matures, the degree to which it is a suitable host plant for *B. tabaci* changes. There are likely to be several factors associated with the ageing process such as changes to leaf morphology, plant biochemistry and *B. tabaci* preference and learning that impact this process. The population of *B. tabaci* builds up starting at three MAP and peaks between five and seven MAP (Sseruwagi *et al.*, 2003), when the foliage is very well formed and succulent after which it drops drastically as the plants grow taller, become more woody (less succulent) and shade the leaves. However, overall, the dynamics of *B. tabaci* populations in the field in response to factors that change as cassava ages have not been well documented.

All the cultivars surveyed in Uganda in 1990–1992 were susceptible to CMD (Otim-Nape *et al.*, 1998), but as cassava plants age, the rate at which the CMD spreads is reduced (Fargette *et al.*, 1993). Cuttings taken from the top of the plant are more likely to be virus-free for CBSD compared with those taken from the bottom of the plant (Mohammed *et al.*, 2016), which may be related to plant age. During sampling for virus detection, virus titre is always highest in the older leaves for CBSVs, especially in the young (<6 months old) cassava plants. Research to identify the resistance mechanisms in cassava cultivars shows that some cultivars can recover as the plants age (known as reversion, Adriko *et al.*, 2011). CMD symptoms disappeared and cuttings taken from initially infected plants developed without disease symptoms (Gibson & Otim-Nape, 1997; Adriko *et al.*, 2011).

#### Cassava virus infection status

There are some empirical studies that have tested the hypothesis that there is a relationship between disease severity in a plant and *B. tabaci* abundance (Gregory, 1948; Leuschner, 1977; Robertson, 1987; Fargette *et al.*, 1993; Otim-Nape *et al.*, 1995; Colvin *et al.*, 2004). If this is due to correlation or causation it is often hard to untangle. The abundance of *B. tabaci* adults was shown to be significantly higher on healthy cassava plants compared with infected plants, but adults stayed longer on diseased plants and aggregated on the green plant tissue. This resulted in higher density of adults by photosynthetic leaf area (area of living leaf tissue) compared with plants without disease. Omongo (2003) posits that this increased density might trigger the adults to disperse. Results also show that adults are more likely to move from clean to infected plants, and diseased plants increased fecundity (Omongo, 2003).

Cassava plants infected with CMBs have been reported to be more suitable for growth and development of B. tabaci. A summary of the studies showing the effect of virus infection of host plants on *B. tabaci* population growth, development and behaviour can be found in Colvin et al. (2006). Concentrations of amino acids have been shown to be greater in infected cassava, and these may benefit *B. tabaci* fitness (Colvin *et al.*, 1999, 2006). However, other laboratory studies have found that the status of cassava disease and *B. tabaci* (i.e. viruliferous or non-viruliferous) had no significant effect on life-history factors, sex ratio and developmental period, or per cent adult emergence (Thompson, 2011). Additionally, the longevity of *B. tabaci* was shown to be reduced when they carry viruses such as *tomato yellow leaf curl virus* (Berlinger *et al.*, 1996). Therefore, whilst infection status plays some role in altering the bottom–up resources for *B. tabaci*, we cannot say when and how this will lead to high abundance in a field situation.

#### Non-cassava host plants

Bemisia tabaci is a polyphagous herbivore that can potentially use a wide range of different host plants in cassava production landscapes. Evidence from outside of Africa (Bellotti *et al.*, 2005) and from West Africa (Burban *et al.*, 1992) shows that *B. tabaci* can have very different associations with different host plants in different locations indicating the likelihood of host-plant associated genotypes. Research in West Africa showed two genotypes of B. tabaci; one polyphagous on a range of plants (excluding cassava) and the second found only on *Euphorbia* species (this group includes cassava) (Burban *et al.*, 1992). Laarif et al. (2015) found that *B. tabaci* Mediterranean (MED, formally named biotype Q) preferred host plants in the families Verbenaceae and Malvaceae, and Middle East-Asia Minor 1 (MEAM1, formally named biotype B) were found on Cucurbitaceae and Solanaceae. SSA2 only occurred on *Datura* and eggplant (Laarif *et al.*, 2015). Their results support the argument that the genetic differentiation of *B. tabaci* species does not operate at the plant species level, but more likely in response to broader taxonomic grouping, for example, plant families. Table 4 documents host plants that have been recorded in recent publications that included a genetic determination of the species. Most of the studies rely on adults (which are highly mobile) recorded on host plants, except Sseruwagi et al. (2006) who used nymphs to confirm the results obtained with adults for host-plant colonization. There is a supposition that the number of eggs laid on a plant is a better indicator of a preferred host compared with counts of adults (Laarif *et al.*, 2015). Further information is required that shows clear species–host-plant relationships in field contexts, such as preference tests, rate of nymphal development and mortality on host plants (not just presence or absence).

**Table 4:** Host plants of *Bemisia tabaci* in East Africa from the published literature.

Host plant	Common name	*B. tabaci* genotype^*^	References
Manihot esculenta	Cassava	Ug1, Ug2, SSA1, IO	Sseruwagi *et al.* (2005); Tajebe *et al.* (2015)
Ocimum gratissimum	Wild basil	Ug3	Sseruwagi *et al.* (2005)
Cucurbita pepo	Squash	Ug4, MED, EA1	Sseruwagi *et al.* (2005); Tajebe *et al.* (2015)
Cucurbita sativus	Cucumber	Ug4	Sseruwagi *et al.* (2005)
Leonotis nepetifolia	Klip dagga, Christmas candlestick or lion's ear	Ug4 EA1, MED, IO	Sseruwagi *et al.* (2005); Tajebe *et al.* (2015)
Pavonia urens	Malvaceae, hibiscus-like flower	Ug4	Sseruwagi *et al.* (2005)
Commelina benghalensis	Wandering jew, or benghal dayflower	Ug7	Sseruwagi *et al.* (2005)
Phaseolus vulgaris	Bean	Ug7	Sseruwagi *et al.* (2005)
Abelmoschus esculentus	Okra	Ug1, Ug6, EA1	Sseruwagi *et al.* (2005); Tajebe *et al.* (2015)
Lycopersicon esculentum	Tomato	Ug1, Ug8, SSA1, IO	Sseruwagi *et al.* (2005); Tajebe *et al.* (2015); Delatte *et al.* (2011)
Gossypium hirsutum	Cotton	Ug8, EA1	Sseruwagi *et al.* (2005); Tajebe *et al.* (2015)
Ipomoea batatas	Sweet potato	Ug1, EA1, MED, SSA1	Tajebe *et al.* (2015)
Solanum melongena and Datura sp.	Eggplant	SSA1 (very few specimens), Tunisia	Laarif *et al.* (2015)
Euphorbia heterophylla, Aspilia africana	Non-crop weeds	Ug1	Sseruwagi *et al.* (2005)
Manihot glaziovii Jatropha gossypifolia	Tree cassava	Ug1	Sseruwagi *et al.* (2006)
Lantana spp.	Lantana and hibiscus	MED, in Tunisia	Laarif *et al.* (2015)

* The names used here are the same as authors used in their papers, however see section on species identification.

Experiments transferring *B. tabaci* from natal host plants to different local host plants result in failure or variable establishment. These results were used to support the idea that there are different *B. tabaci* genotypes with restricted host ranges (Burban *et al.*, 1992). However, this research did not test the influence of host-plant transfer on ability of *B. tabaci* to transmit disease. Research by Antony et al. (2006) showed that natal host plants influence the ability of *B. tabaci* to transmit *Indian cassava mosaic virus* (ICMV). Whereas *B. tabaci* reared from cassava could transmit ICMV to cassava, *B. tabaci* reared on sweet potato were unable to transmit ICMV to cassava. There was a significant difference in the presence of the cyanide detoxifying enzymes in cassava reared *B. tabaci* compared with those reared on sweet potato. Together, the results show the ability of *B. tabaci* to adapt to different host plants.

Intercropping cassava with other crop plants (e. g. coffee, maize, sweet potato, bean, groundnut) is common practice in many parts of East Africa. However, beyond saying if a crop is likely to be a host plant or not, we cannot yet make recommendations about which intercrop would be most useful for reducing *B. tabaci* abundance on cassava. Intercropping cassava with maize was shown to reduce *B. tabaci* population abundances in the Ivory Coast (Fargette *et al.*, 1988), although the mechanism here may not be related to host-plant preferences, but rather host-plant availability and physical barriers (i.e. maize are not host plants and may create a barrier to accessing host plants). Intercropping cassava with cowpea has been shown to decrease numbers of *B. tabaci* in Colombia (Gold *et al.*, 1989). Results of surveys in Uganda in 2007 showed that intercropped cassava had significantly less *B. tabaci* than monocrops (Night *et al.*, 2011). Experiments intercropping cassava with *Vigna unguiculata* and *Vigna radiata* (cowpea and green gram mung bean) showed reduced *B. tabaci* populations and severity of CMD. Disease-free cuttings of two cultivars (one susceptible local cultivar and one improved cultivar) were used in field experiments. Compared with monocrop treatments, the cultivars intercropped with mung bean had significantly less *B. tabaci* and disease incidence and severity for both the local and improved cultivar (Uzokwe *et al.*, 2016).

#### Spatial and temporal arrangement of host plants

As well as the influence of intercropping per se on *B. tabaci* populations in cassava fields, the spatial and temporal arrangement of crops and other potential non-crop hosts around cassava fields may also influence population growth and abundance in the crop field, especially early in the growing season. In theory, if host plants surrounding cassava fields facilitated the early arrival (and high numbers of colonizers) of the first generation of *B. tabaci* into the cassava field in the early stages of the crop, this may lead to an outbreak. Furthermore, if the spatial and temporal arrangement of host plants negatively impacted the dynamics of natural enemies of B. tabaci, this could also lead to an outbreak.

In a farming landscape where a species of *B. tabaci* (MEAM1) has been shown to be polyphagous with several crops and wild host plants suitable to support population growth (Queensland, Australia, Sequeira *et al.*, 2009; De Barro, 2012), it was possible to develop a landscape model to simulate how the spatial and temporal arrangement of host plants influences *B. tabaci* abundance and ‘outbreaks’. The model simulations indicated that peak densities of MEAM1 *B. tabaci* were higher for low or non-suitable crops than for crops with a medium suitability. This counter-intuitive result was explained by the fact that medium suitability winter crops supported high parasitoid (*Eretmocerus hayati*) populations, which can suppress *B. tabaci* populations in summer crops (De Barro, 2012; Kristensen *et al.*, 2013). Therefore, both the surrounding landscape and crop rotation choices had a significant effect on simulated *B. tabaci* population dynamics.

Understanding how the farming landscapes in East Africa offer resources for both *B. tabaci* and its natural enemies is challenging due to the variegated nature of the land-use patterns characteristic of smallholder farming. Often there are multiple crops planted in each field or garden and rotation practices are flexible and dependent on the family, village and regional demand for certain food types. However, studies to quantify the effect (even if small) of the spatial and temporal arrangement of host plants are needed because this knowledge may lead to easily adoptable changes in management practices.

#### Natural enemies

Breeding cassava cultivars that are resistant to disease has been the main approach used to manage epidemics of CMD. However, as part of an integrated management plan to control B. tabaci, identifying ways to enhance naturally occurring predators and parasitic wasps also needs to be considered (Legg *et al.*, 2003). Fishpool & Burban (1994) noted that there were 30 parasitoids of *B. tabaci* worldwide, and 40 generalist predators. However, the ecology and impact of parasitoids and predators of *B. tabaci* in East Africa remains relatively unknown.

Regarding predators, Phytoseiidae mites, such as *Euseius scutalis*, have been recorded predating *B. tabaci* populations on cassava in Kenya (Otim-Nape *et al.*, 1995), and mirids, such as Nesidiocoris tenuis, have predated *B. tabaci* on other crops such as tomato (Calvo *et al.*, 2012). Results from petri dish experiments with *B. tabaci* from cotton showed that the predatory mite *Amblyseius aleyrodis* Elbadry readily consumed *B. tabaci* eggs in a no-choice environment (Elbadry, 1968). Similarly, from the work carried out in the USA, *Euseius hibisci* were shown to consume and complete their development on *B. tabaci* (Meyerdirk & Coudriet, 1985). Other predators of *B. tabaci* nymphs from around the world include Stethorus jejunus Casey, Coccinellidae, *Holoborus pallidicornis* (Cameron) Staphylinidae and *Scolothrips latipennis* Priesner, Thysanoptera (Fishpool & Burban, 1994). The Neuropteran *Conwentzia africana* Meinander is considered an important predator of *B. tabaci* (Legg *et al.*, 2003). *Serangium* sp. (Coleoptera: Coccinellidae) can complete their development feeding on juvenile stages of *B. tabaci* on cassava (Asiimwe *et al.*, 2007a, b). No-choice laboratory experiments showed that *Serangium* larvae could consume over 1000 nymphs in total. The maximum number of nymphs consumed per day was mid-way through their development, when *Serangium* larvae consumed over 200 nymphs per day (Asiimwe *et al.*, 2007a, b). We know that cultivars of cassava with different morphologies can influence the activities of predators such as *Typhlodromalus aripo*, the mite that preys on the pest CGM *M. tanajoa* (Zundel *et al.*, 2009).

Legg & Hillocks (2003) lists the parasitoids attacking *Bemisia* genus in SSA. Thirty-four species of *Encarsia* and 14 species of *Eretmocerus*, with *Eretmocerus mundus* Mercet and *Encarsia sophia* Girault and Dodd being the most dominant (Legg *et al.*, 2003). Surveys of *B. tabaci* parasitoids in cassava in Tanzania identified using a molecular approach, ten species of parasitoids (Guastella *et al.*, 2015). Hoelmer et al. (1995) summarized several papers that suggested that parasitoids may be insufficient to control *B. tabaci* without other control methods. However, parasitism rates of up to 58% have been recorded in Uganda (table 5). Some work has been completed to quantify the impact of parasitoids on B. tabaci.

**Table 5:** Records of parasitism of *Bemisia tabaci* from field studies in EastAfrica.

Citation	Location/study type	Host plant	Parasitoid species recorded	Percentage parasitism
Otim et al. (2005)	Namulonge, Uganda. Survey data	Cassava with *B. tabaci*	*Eretmocerus mundus* *Encarsia mineoi* *Encarsia Sophia* *Encarsia* ‘blackhead’ (undescribed)	40-58%
Otim et al. (2006)	Namulonge, Uganda. Field study on cassava cultivars	Cassava with *B. tabaci*	*E. mundus* *E. sophia*	20-58%
Otim et al. (2008)	Namulonge, Uganda. Potted plant study	Cassava potted plants with *B. tabaci*	*E. mundus* *E. sophia*	11–67% hirsute cultivar 0–42% glabrous (trial 1) 0–46% hirsute cultivar 0–67% glabrous (trial 2)
Guastella *et al.* (2015)	Mwanza, Shinyanga and Tabora, Tanzania. Survey data	Cassava	*E. Sophia* *En. Guadeloupae* *En. Dispersa* *En. Lutea* *En. mineoi* *En. sp. pr. circumsculpturata* *Er. Mundus* *Er. sp. pr. hayati or Queenslandensis* *Er. sp. 1* *Er. sp. 2*	Parasitism levels not determined

*Eretmocerus mundus* and *E. sophia* were shown to parasitize *B. tabaci* on cassava in Uganda and accounted for 34% parasitism of fourth instar nymphs (Legg, 1995). Significantly higher number of *B. tabaci* and parasitoids occurred on the CMD-resistant cultivar compared with a susceptible cultivar although parasitism rate was similar. Although not tested for specifically, the cultivar and presence or absence of CMD did not seem to influence parasitism rates. Per cent parasitism was recorded as <20%, and on three occasions <50%. However, results showed a significant negative relationship between parasitism rate and nymph numbers indicating that these parasitoids did not respond in a density-dependent manner (Otim *et al.*, 2006). Life-history studies conducted under field conditions showed that dislodgement was the key mortality factor for eggs and that parasitism (mostly by *E. sophia* and *E. mundus*) caused the highest mortality to fourth instar nymphs. There was no difference in results from the treatments exposed to, or sheltered from, the rain (Asiimwe etal., 2007a, b).

There has been little research to understand how different cassava cultivars might influence the activities of natural enemies of *B. tabaci*. We know that cultivars of cassava with different morphologies can influence predators such as *T. aripo* (the mite that preys on *M. tanajoa*, Zundel *et al.*, 2009), and there have been some basic experiments conducted using parasitoids (Otim *et al.*, 2008). However, a comprehensive understanding of cultivar impacts at higher trophic levels is critically needed.

#### Competition with other herbivores on cassava

Competition between *B. tabaci* and other herbivores on cassava may impact the abundance of *B. tabaci*. For example, the CGM *M. tanajoa* is often found on the top leaves of the cassava plant, making these leaves less suitable for *B. tabaci* adults (Legg *et al.*, 2015). Interspecific interactions between pests on the same crop can significantly influence invertebrate behaviour and host-plant defences; for example, the duration and density of the aphid *Myzus persicae* on tomato significantly affected the number of *B. tabaci* (Tan *et al.*, 2014). We could find no studies that examine the interactions between the community of pest and non-pest herbivores on cassava in East Africa.

#### Endosymbionts

Some evidence exists that endosymbiotic bacteria within *B. tabaci* can have both positive and negative effects on *B. tabaci* fitness (Kontsedalov *et al.*, 2008; Himler *et al.*, 2011; Ghosh *et al.*, 2015). *Portiera aleyrodidarum* is a primary obligate bacterial endosymbiont of B. tabaci, and is essential to their development. As well as obligate bacteria, they have an association with many facultative bacteria or secondary endosymbionts. In theory, these bacteria may confer some advantage for transmission of CMBs by *B. tabaci* and help them adapt to new host plants (Gottlieb *et al.*, 2010; Kliot *et al.*, 2014).

The association between facultative secondary endosym- bionts and various species of *B. tabaci* was explored using samples collected in Tanzania from cassava and adjacent host plants, mostly crops and one weed (Tajebe *et al.*, 2015, see graphic depicting relationships between different groups of *B. tabaci* such as SSA1-SG1). Most *B. tabaci* collected from cassava were SSA1 and most were uninfected by any of the secondary symbionts. A later study found contrasting results (Ghosh *et al.*, 2015). Samples of *B. tabaci* were collected from cassava crops across East African countries were found to be infected with a range of endosymbionts, with the predominant species being *Wolbachia*, *Rickettsia* and *Arsenophonus*. The prevalence of these secondary endosymbionts including *Wolbachia* varied characteristically across each *B. tabaci* population (Ghosh *et al.*, 2015). Association of the endosymbionts varied across geographical boundaries and the *B. tabaci* species. SSA1-SG3 in coastal Eastern Africa had high levels of *Arsenophonus* and *Rickettsia* in single or mixed infections (84%), while a small proportion (13%) was free of detectable secondary endosymbionts (Ghosh *et al.*, 2015). In contrast, SSA1-SG1 collected in the highland regions of Uganda and around Lake Victoria had different secondary endosymbiont profiles. About 25% of SSA1-SG1 individuals were infected with *Arsenophonus* and *Rickettsia* in single or mixed infections, while equal proportion of endosymbiont-free (38%) and *Wolbachia*-infected individuals (37%) were found in Uganda. In laboratory studies, all three bacteria (*Wolbachia*, *Arsenophonus* and *Rickettsia*) were shown to negatively impact *B. tabaci* population development by reducing adult emergence and simultaneously increasing nymph development time, thereby reducing number of adults and the number of generations that can be developed per unit time (Ghosh *et al.*, 2015). In addition to several factors discussed above, it has been proposed that high levels of bacteria-free *B. tabaci*, which are fitter and more fecund, may have contributed to high abundances in certain regions. Similar effects have been observed in *Drosophila* and mosquitoes infected with *Wolbachia* (McMeniman & O’Neill, 2010). Thus, it is possible that the negative effects of endosymbionts in *B. tabaci* have been important population control mechanisms in these regions.

### Abiotic factors

#### Altitude

There is evidence in the literature that altitude relates to population abundance of B. tabaci. However, the mechanism underlying any altitudinal variations seen in the few studies available (e. g. temperature, rainfall gradients, change in farming systems and crops grown) have not been tested (or in some cases even described). There is some evidence to suggest that cassava virus infection was lower in areas above 800 m above sea level (Legg (1994)). Legg & Raya (1998) found a significant negative correlation between CMD incidence and altitude in Tanzania. Historically, it has been noted that at high altitudes (>1000 m above sea level), there are less plant disease problems and an absence of *B. tabaci* in cassava, presumably due to cold temperatures. In general, there is evidence of a trend of declining CBSD incidence with increasing altitude in the coastal zone of Tanzania, but not in the lake zone (Jeremiah *et al.*, 2015).

#### Climate and weather

As with all invertebrate pest species, long-term climate patterns and short-term weather events will influence population growth and development of B. tabaci. However, drawing conclusions beyond general statements is challenging due to a lack of information for the species associated with cassava in East Africa. In general, *B. tabaci* populations are favoured by high temperatures and moderate rainfall (Sseruwagi *et al.*, 2004). Robertson (1987) described increases in the abundance of *B. tabaci* along coastal Kenya related to an increase in annual rainfall, and increased activity of flying adults after the end of rainy periods. Recent analyses of *B. tabaci* adult abundance and environmental factors have shown that abundance was higher with high minimum temperatures and lower mean annual rainfall in the coastal zone of Tanzania (Jeremiah *et al.*, 2015). However, in the lake zone of Tanzania, mean annual rainfall and the length of the growing season were the most important environmental factors. Some studies note generally when numbers of *B. tabaci* are likely to be low in cassava fields based on the time of the year when temperatures are low and the environment is unsuitable for *B. tabaci* (Mbewe *et al.*, 2015). At a finer scale, we know that micro-climate variability within a field can influence the numbers of *B. tabaci* found on cassava plants. *Bemisia tabaci* adults decrease as planting density decreased and canopy temperatures increased (Otim-Nape & Ingroot, 1986).

If we examine studies that include *B. tabaci* species more broadly (i.e. not just East African studies), humidity extremes (low humidity <20% and high humidity >80%) can increase mortality of immature stages, and development rate of multiple life stages decreases dramatically with temperatures above 30–33°C (Gerling *et al.*, 1986). Drost *et al.* (1998) used an upper lethal temperature of 36°C to fit a development rate model for immature *B. tabaci* on cotton. Laboratory studies have shown that *B. tabaci* survival ranges from ~90% survival at 25°C and 100% RH, to <2% survival at 41°C and 20% RH (during a 2 h exposure) (Berlinger *et al.*, 1996).

### Other factors and hypotheses

#### Pesticides

The overuse of pesticides and rapid development of resistance in *B. tabaci* has been shown to cause high abundance and change the identity of the common *B. tabaci* species in other cropping systems around the world (e. g. Crowder *et al.*, 2008). For example, a shift from *B. tabaci* MEAM1 species to MED species was found in cotton fields in Israel and this change in species composition had an impact on resistance to insecticides, with one population showing less resistance to insect growth regulators (Horowitz & Ishaaya, 2014). However, the use of pesticides by East African smallholder farmers has historically been low due to their cost and availability, although their use is increasing each year (de Bon *etal.*, 2014). Insecticide application in cassava production landscapes in East Africa is limited to crops such as tomatoes and other fruit and vegetables (de Bon *et al.*, 2014). Documented statistics on pesticides use (and especially insecticide use) patterns in cassava by smallholder farmers in East Africa is rare. Surveys of honeybee hives throughout Kenya showed low levels of pesticide contamination in the hives (Muli *et al.*, 2014). Documentation of the change in insecticide use patterns over time (products, active ingredients, crops, application rates and baseline levels of resistance) may help predict the onset of resistance development and help in the development of an integrated resistance management strategy.

#### A new invasive species in East Africa

Given the confusion surrounding the taxonomy of species in the *B. tabaci* complex, we cannot rule out that there have been one or multiple incursions of an entirely new species into this region over the recent historical period. As an analogous example from outside of East Africa, the exotic pest B. ta- baci MEAM1 was first detected in Australia on ornamental plants in 1994, but it was not until 2001 that high numbers on fruit and vegetable required control (Gunning *et al.*, 1995; Sequeira *et al.*, 2009). After this new species entered East Africa, it may have been better able to exploit resources in cassava production landscapes, avoid attack by natural enemies, and outcompete domestic *B. tabaci* species. In addition to natural spread within the African continent, movement of species into new areas is possible via human-assisted transport (Caciagli, 2007). Yet there is no empirical evidence to support this idea in East Africa (table 1).

#### Hybridization

The *B. tabaci* abundance associated with the spread of the severe CMD pandemic in Uganda in the late 1990s was believed to be due to the appearance of an invasive SSA2 *B. tabaci* species (Legg *et al.*, 2002; ). However, subsequent studies by Sseruwagi (2005) and Mugerwa et al. (2012) showed SSA2 to be less abundant in Uganda post-invasion. Instead, the areas with high *B. tabaci* populations had a distinct clade of SSA1 (SSA1-SG1), and what was believed to be a hybrid of SSA2 and SSA1. More recently, Tajebe *etal*. (2015) also suggested hybridization as the underlying cause in the change from *B. tabaci* SSA2 to *B. tabaci* SSA1-SG1 in Tanzania, and that the CMD pandemic was now associated with high abundances of *B. tabaci* SSA1-SG1 genotype. However, empirical studies to confirm this hypothesis in East Africa have not yet occurred.

Empirically detecting such changes in field studies on a pest complex can be very challenging (but not impossible, see discussion in Liu *et al.*, 2012). The process of hybridization is unlikely to be reflected by the mtDNA COI gene currently used for identification purposes. Given the mitochondrial DNA genome’s overall maternal inheritance property and its general lack of recombination hybridization between a population carrying the SSA2 mtDNA COI haplotypes with the SSA1 mtDNA COI haplotypes would result in the hybrid off spring being either SSA2 or SSA1 mtDNA COI haplotypes, but is unlikely to generate the SSA1-SG1 mtDNA COI haplotype signature. To show evidence of hybridization, we need to focus on changes in patterns in the nuclear genome, and then link these patterns with ecologically relevant fitness traits that may increase population growth and abundance on cassava.

#### Knowledge gaps

Given that many of the factors that potentially influence *B. tabaci* abundance listed in table 1 have had very little research surrounding them in East Africa, and may interact with each other in antagonistic or synergistic ways; therefore, identifying which are the critical knowledge gaps is challenging. Our focus here is on identifying knowledge gaps, which if filled, may lead to more sustainable and durable solutions to *B. tabaci*-associated crop damage in East Africa. Underpinning all the knowledge gaps highlighted below is the species identification issue. Without well-documented species nomenclature, set within a robust framework for identifying new species, the biological and ecological information generated may be lost rapidly. The high priority knowledge gaps are outlined below.

#### *Which East African* B. tabaci *species commonly use cassava as a reproductive host plant?*

Whilst *B. tabaci* adults are highly mobile and can be found on a number of plants, establishing which species commonly use cassava as a reproductive host plant (i.e. they can oviposit and complete nymphal development) is important. It is these species for which we need to devise targeted management interventions to control. To address this research question requires the identification of large numbers of field-collected nymphs using nuclear molecular markers, and reciprocal crossing experiments using cultures developed from nymphs reared through to adults. Laboratory studies looking at basic life-history parameters of the different species under different temperatures and humidities could then be conducted. This is also the first step in establishing if these target species also use alternate host plants besides cassava.

#### *To what extent do non-cassava host plants contribute to the population dynamics of* B. tabaci *and the spread of cassava diseases?*

Whilst establishing the diversity of potential host plants that can be used by *B. tabaci* in production landscapes is important, we must take this one step further and establish if, when and how, these alternate host plants impact *B. tabaci* abundance and disease spread in cassava crops. For example, can alternate host plants for *B. tabaci* serve as reservoirs of viruses that may be transmitted to cassava (Alabi *et al.*, 2008)? If an alternative host plant is identified, but is relatively rare in the landscape, will it impact the population dynamics in cassava? Conversely, if an alternate host plant is common in the landscape, will its removal impact population dynamics in cassava? There are straightforward management recommendations that can be developed from improved understanding about alternate host plants and the role they play in an agricultural landscape.

#### How does the proportional availability of infected vs. uninfected cassava plants in a landscape influence disease risk and spread?

It has been suggested that *B. tabaci* shows preferences for infected cassava plants, and infection can alter the performance of *B. tabaci* at the population level. However, we do not understand how this manifests in real cassava production landscapes, with a diversity of cassava cultivars, showing different levels of disease. Modelling the spread of CMD via infected cuttings assuming that *B. tabaci* prefer infected over uninfected plants, in combination with the proportion of infected plants available, indicated this could have major implications for disease spread. Incorporating information at a landscape scale about which species of *B. tabaci* are efficient vectors of each virus would also improve model predictions. Extending this to a detailed quantification of yield loss due to cassava diseases in the presence and absence of *B. tabaci* at the field and landscape level is also necessary to inform future management options.

#### *How can we use choice of cassava cultivars in production landscapes to reduce population abundances of* B. tabaci?

Besides establishing the effect of different cassava cultivars on the fitness and performance of B. tabaci, we need to provide recommendations that lead to population reductions or lower risk of outbreaks at the landscape level. An understanding of the relationship between disease dynamics across a landscape, *B. tabaci* movement between cultivars, and cultivar diversity and abundance is needed. From this understanding, we may be able to provide location-specific recommendations about the selection of ideal cultivars, guidance on rouging and cassava-free periods. Historically, the adoption of new and improved cassava cultivars has been variable within countries, so more effort to understand the best mechanisms for ensuring that the new cultivars that are adopted also lead to *B. tabaci* population reductions would be valuable.

#### *What is the impact of natural enemies in East Africa on* B. tabaci *and can they reduce the risk of outbreaks?*

Whilst we know there are a diversity of natural enemies present in cassava fields that can cause mortality of B. tabaci, we cannot say what role these species play in reducing the frequency or likelihood of *B. tabaci* outbreaks (and if this will impact disease outbreaks). Given that cassava is a crop with a relatively long growth season (compared with many vegetables), and now receives relatively little pesticide applications, it is important that we explore further the potential impact of natural enemies. Furthermore, the integration of natural enemies with other management options (e.g. host-plant resistance and habitat management) is critical.

There is very little information about the natural enemies that prey on different stages of *B. tabaci* in field conditions and the impact they have on B. tabaci. Therefore, there is a need to better understand their biology and behaviour (life history of individual species), their relationships and interactions with other predators and parasitoids, and quantify the impact they have on *B. tabaci* populations. For some groups, we lack fundamental information on whether they frequently predate on B. tabaci. For other factors, such as the effect of alternative host plants (i. e. do any provide an alternative source of natural enemies to recolonize cassava crops and attack B. tabaci), dispersal ability, response to semiochemicals, and methods to increase fitness and population growth need to be determined. It is important to quantify the scale at which natural enemies may have an impact (i.e. within a few tens of metres or within 100 m of a source field), to enable us to make specific management recommendations to farmers.

#### *How can we sustainably manage the use of insecticides in East Africa to delay or avoid resistance in* B. tabaci?

If insecticide use increases in the coming years, such as in vegetable crops in or near cassava, or in cassava itself, there is the potential for *B. tabaci* species attacking cassava to be exposed to strong resistance selection pressures. Experiences in cotton production landscapes elsewhere have shown that resistance can develop quickly in *B. tabaci* (Crowder *et al.*, 2008; Gnankine *et al.*, 2013) and studies should consider establishing baseline levels of resistant alleles in populations now. Furthermore, the testing and development of products based on newer chemistries, which have less non-target impacts, needs to be conducted in East Africa.

#### What research methodologies do we need to develop now to enable scientists to ask the right questions in the future?

Throughout this review, we have highlighted methodological limitations that restrict research and the questions that scientists can address. For example, we need a smarter way of estimating *B. tabaci* adult numbers in fields with high abundances. In cases where nymphal or egg data may provide a more informative picture of a certain ecological process, counting adults could be avoided. We can develop new and fast approaches to count, collect, record and identify nymphs if that is what is needed to address a research question. A field-based method that allows us to separate virus infection borne by B. tabaci, from that borne by cuttings (or a combination of both agents) would greatly aid in our understanding of *B. tabaci* as a vector (see an example in Tajebe *et al.*, 2015). A rapid diagnostic test for virus infection at the cutting stage would enable researchers to decide which factors they wanted to examine in their study, and be confident of their results. In addition, the advent of an infield diagnostic strip would allow scientists to detect virus at a given period and easily map patterns of disease spread. In another example, the recent development of a transcriptome technique that can provide data from one *B. tabaci* individual by Sseruwagi et al. (2017 submitted) will reduce reliance on the use of isolines for transcriptomics studies, and could therefore help to resolve some of the urgent questions about the biological differences between *B. tabaci* species.

#### What are the economic trade-offs associated with different management options for smallholder farmers, and what networks need to be available to support adoption?

Fundamental to the deployment of new management interventions, and adoption by farmers, is strong extension networks with smallholder farmers and the wider cassava value-chain actors. Without this network, the adoption of durable solutions to *B. tabaci* control will be slow or unlikely to occur. Furthermore, a complete economic assessment of the trade-offs for smallholder farmers associated with adopting different practices is needed to ensure that management options are set in the current-day economic realities of these farmers. Often, researchers spend a lot of time understanding the biophysical constraints on a system but neglect the linked socio-economic system in which farmers operate. To bring about change in how this pest is managed in the future, we need to assess both systems at the same time.

## Conclusions

Given the right combination of environmental factors, many species of *B. tabaci* within the complex have the potential to become a pest at any one point in time and exhibit outbreaks in certain locations. Furthermore, these critical factors may vary from country to country and even region to region across East Africa. Our challenge is greater than just identifying factors; we must go one step further and identify which factors are the most important for smallholder farmers to manage to minimize the risk of outbreaks. This review represents a comprehensive summary of the knowledge to date, and should be used to guide future research questions by scientists all over the world addressing this challenge.
